# Machine learning applications in sport: a scoping review

**DOI:** 10.3389/fpsyg.2026.1802549

**Published:** 2026-05-25

**Authors:** Antonia Cattle, Kathryn Johnston, Alexander B. T. McAuley, Adam Kelly, Joseph Baker

**Affiliations:** 1 Tanenbaum Institute for Science in Sport, University of Toronto, Toronto, ON, Canada; 2 Research for Athlete and Youth Sport Development (RAYSD) Lab, Research Centre for Life and Sport Sciences (CLaSS), School of Health Sciences, Birmingham City University, Birmingham, West Midlands, United Kingdom

**Keywords:** artificial intelligence, deep learning, machine learning, neural networks, sport

## Abstract

Machine learning (ML) applications continue to grow in popularity across the sport industry, offering new opportunities for performance enhancement, injury prevention, and decision-making. The present scoping review examined the landscape of ML applications in sport by analyzing 270 peer-reviewed studies published between 2002 and 2024. ML was applied across 12 broad subject areas, with computer science, biomechanics, and sport psychology emerging as the most common domains of application. Key applications included action recognition, injury prediction/prevention, and athlete selection/talent identification. While ML models have demonstrated promising accuracy, their practical utility was often limited by issues of data quality, interpretability, and accessibility for end users such as athletes, coaches, and sport interest-holders. Given the issues surrounding the practical usability of ML, the ultimate goal of ML should be to support – not replace – human expertise, as its integration may enhance sport and athlete experience at all levels of engagement and development.

## Introduction

From using global positioning systems to find the best route to your destination, to tracking activities with wearable technologies (e.g., smart watches), artificial intelligence (AI) technologies are now seamlessly integrated into daily routines. Alongside this widespread adoption in daily contexts, AI has gained significant traction in the realm of sport. A high-profile example is Major League Baseball’s introduction of the automated strike zone system, or “robo umps”, currently in its first year of use in major league baseball. Similarly, the National Football League (NFL) is preparing to replace the traditional “first down” chain with an AI-driven Hawk-Eye (i.e., video surveillance and measurement system) camera – a change expected to enhance accuracy and game pace by saving up to 40 s per play (ESPN, 2025). In Formula 1 racing (F1), teams increasingly employ AI to forecast weather, optimize pit stop strategies, and predict competitor behaviour (Marr, 2023). F1 has also started using simulators powered by neural networks (NN) to train drivers off track, reducing costs and risks. These technological developments reflect a growing movement to reduce human error and increase efficiency through AI. While AI is reshaping how sports are played, analyzed, and experienced by athletes, coaches, officials, and fans, its adoption has not been without controversy and resistance. For instance, in baseball, some have described the introduction of these automated officiating systems as “…one of the most drastic structural changes in the Major Leagues…”, and while praised for improving consistency of strike and ball calls, these systems have also drawn criticism from both fans and officials (Perry, 2024). Similarly, critics of F1’s technological expansion argue these changes come at the expense of the sport’s cultural identity (Marr, 2023).

Despite the breadth of applications, the term ‘artificial intelligence’ often masks the complexity of the underlying technologies. This review focuses on Machine Learning (ML) – a branch of AI that allows systems to learn from data, adapt over time, and generate predictions or decisions. ML can be seen as a dynamic AI, where it continues to learn through patterns in input data, similar to how humans learn information, through inductive learning (Chmait and Westerbeek, 2021). ML represents the dominant and most widely used branch of AI in sport contexts and is the focus chosen for this review. While these technologies offer benefits like efficiency and personalization, their practical use may not live up to expectations. For instance, ML methods used for injury prediction show promise for identifying athletes at elevated injury risk, but the overall methodological quality of this work is low and clinical applications have been hindered by wide prediction windows and broad injury definitions (Claudino et al., 2019; Leckey et al., 2025; Pillitteri et al., 2023; Van Eetvelde et al., 2021). Beyond athlete monitoring, ML is being used to promote fairness in judged sports. At the 2023 Gymnastics World Championships, for example, Fujitsu’s computer vision based judging support system aimed to reduce bias by providing “unbiased and error free” scores (Mazurova et al., 2022; Taylor Price, 2024). However, such systems struggle to capture the artistic and expressive elements central to many sports, raising concerns about diminishing the richness and authenticity of the sport.

AI is also being used in the areas of talent identification, athlete development, and performance prediction. In the past, talent identification heavily relied on the capabilities and intuition of coaches and scouts. Now AI has the ability to analyze player performance data, physical metrics, and even psychological traits for athlete selection purposes (McAuley et al., 2024). A recent study examined how baseball players’ strength, speed, and anthropometric data could be used to predict in game batting performance. The authors used a random forest regression coupled with a feature importance analysis to identify physical traits that were most influential in predicting batting outcome (i.e., speed, strength, and body size), demonstrating how ML can translate these physical attributes into tangible performance insights (Kohn et al., 2024). This work reflects the growing abilities of ML applications, but also highlights critical issues. As Bunker and Susnjak (2022) argue, while ML models show promise in predicting match outcomes, their accuracy depends on the quality of the input data, which continues to be an ongoing challenge. Kilkenny and Robinson (2018) emphasize a foundational rule of statistical analysis – “garbage in = garbage out”; if the data fed into these systems are flawed or biased, any insights produced will be unreliable and/or inaccurate.

Given these strengths and limitations, ML was chosen as the focus of this review because of its capacity to manage complexity, variability, and high volumes of data, commonly encountered in sporting contexts. Moreover, this specific topic was chosen due to its recent application in sport contexts and the growing number of empirical studies in this area. While other reviews on ML and AI applications have used a systematic approach to predict match results, examine athlete biomechanics and performance improvement (e.g., Bunker and Susnjak, 2022; Cruz et al., 2024; Krstić et al., 2023) or focused on specific sports (e.g., soccer; see Pillitteri et al., 2024; Rico-González et al., 2023) or research areas (e.g., injury; see Leckey et al., 2025; Van Eetvelde et al., 2021; Wang, 2024); to the research team’s knowledge, no other review has synthesized the broader landscape to identify key trends and highlight knowledge gaps to facilitate future research avenues. Therefore, the aim of this scoping review was to clarify ML’s evolving role in sport and examine its impact on athletes, coaches, and sport stakeholders. Importantly, researchers must remain mindful of how these tools can complement and support decision-making by providing insights that inform, rather than dictate, actions. This paper explores research across multiple disciplines to highlight: (a) the expanding role of intelligent systems, (b) where challenges persist, and (c) what opportunities may lie ahead.

## Methods

This scoping review followed a 6-step process as outlined by Arksey and O’Malley (2005) and Colquhoun et al. (2014): (1) identifying the research question, (2) identifying relevant studies, (3) study selection, (4) charting the data, (5) collating, summarizing, and reporting results, and (6) consultation. Reporting followed the Preferred Reporting Items for Systematic Reviews and Meta -Analyses extension for Scoping Reviews (PRISMA-ScR) guidelines (Tricco et al., 2018).

### Identifying the research question

After discussions with the research team, we were intrigued by the rapid growth and integration of ML systems in sport. While these technologies can offer new insights and opportunities, they raise questions about reliability, practical applications, and the potential for these technologies to replace or augment human decision-making. With this in mind, the goal for this scoping review was to clarify ML’s evolving role in sport and examine their potential application for athletes, coaches, and sport stakeholders. A secondary goal was to identify the key research areas, highlight areas that may benefit from further investigation, and examine the challenges and opportunities associated with ML technologies and approaches.

### Identifying relevant studies

As this is a continuously evolving field of study, we opted to first conduct a pilot study to gain insight into this field and to test search terms and various databases as a way to refine our search strategy. After reviewing the results of our pilot study and consulting our institution’s research librarian, we determined three preferred databases for our search, including Scopus, SPORTDiscus, and MEDLINE. These databases were chosen due to their extensive indexing and subject headings in sport and ML. Other databases were considered during our pilot study (e.g., IEEE Xplore and Web of Science); however, these were excluded as they have subject and indexing bias (i.e., limited subject coverage, exclusion of grey literature, and missed interdisciplinary work) (van Bellen et al., 2025) and are better used for backward searching (i.e., tracing the evolution of a concept).

Relevant literature was identified using each database’s indexing and search strategy functions. Final search terms were determined based on results from the pilot study, which demonstrated their effectiveness in retrieving articles related to ML in sport while minimizing the number of unrelated articles (i.e., unrelated topics or subject areas). The search terms used were “machine learning”, “deep learning”, and “neural networks.” To ensure inclusion of sport specific content, these ML specific terms were searched in conjunction with “sport”, “sports”, and “sporting” followed by a list of roughly 50 Olympic sports. For an overview of the search strategy, including Boolean search operators, database indexing and subject headings, please see Supplementary material 1.

### Study selection

Once our databases and search terms were selected, the date range of January 1^st^ 1900 to October 17^th^ 2024 was used to search all three databases and the resulting articles were extracted into the reference manager, Covidence^©^.^1^ Two members (AC & SK) of the research team screened for title and abstract eligibility and full text eligibility, using the following inclusion criteria: (1) must include some aspect of ML, deep learning, and/or neural networks; (2) must focus on sport; (3) must include a human athlete population (i.e., not trained robots or digital twins), either as a dataset or a recruited sample; (4), athletes must have been competitive for a minimum of 1 year and levels could range from beginner to Olympic; (5) must be published in English; and (6) must be a peer reviewed journal article, book chapter, or conference proceeding.^2^

Several exclusion criteria were also used to help narrow our full-text inclusions. From our initial list, articles were excluded if they: (1) had un-specified athlete samples or datasets that were not available to confirm the level of the athletes; (2) were methodological or theoretical papers without a sample; (3) only used ML for statistical analysis (e.g., regression analyses conducted in R Studio or Python); (4) focused on E-games, video games, virtual reality, dance, or breaking/break dancing; and/or (5) were a type of review (e.g., scoping, systematic, meta, or narrative), commentary, or full book. If any conflicts arose during the screening process, a third member of the research team (KJ) who was not actively screening, made the final decision on whether to include or exclude the article.

### Charting the data

Once screening was complete, articles were exported into excel for variable extraction, where the primary screener extracted variables of interest for each study. As the goal was to gain insight into the current landscape, in-depth analysis of each study was not necessary. Instead, variable extraction focused on characteristics of value to our review, including country of origin, subject area (broad and specific), type of athlete sample (dataset or sample), sample size, sport, level of athlete (as defined by the authors), and athlete age. These variables were chosen to provide insight into the most popular applications of ML in sport, clarify which areas need more attention, and identify niche applications.

During variable extraction, studies coded as “grey areas” or “tentative inclusion” were discussed amongst the research team. Some examples of articles that fell into these categories and were subsequently removed were those with unclear athlete populations (e.g., indicating they have an athlete population without identifying the sport or level of the athletes), unclear level of athlete, and articles using ‘athlete’ as a descriptor for the sample when discussing participants in physical fitness programs or students in physical education classes. Finally, some articles used unclear datasets taken from previous studies without a description of the population (i.e., level of the athlete and sport[s]). After discussions with the research team these articles were removed prior to variable extraction.

When coding variables, metadata including country of origin by the authorship was identified first. If there were multiple countries in the authorship list, we reported the country of the first author. If there were many authors from the same continent, but from different countries, it was coded as mixed (for example, Europe or North America). When identifying the subject area, the specific topic area was first identified by examining the title and abstract. We then proceeded to identify the broader subject area by determining the primary goal of the article, the academic or medical positions held by the authorship, the journal title/focus, and keywords (e.g., specific area: Orthopedics; broad area: Medicine).

When looking at the sample type used by the authors, if articles specified that they recruited a certain number of athletes or they had collected measures from athletes over time, this type of data was coded as “sample”. When authors indicated they used publicly available data (e.g., player statistics, professional sport data, Olympic results), this type of data was coded as “dataset”. Finally, when authors identified actions or athletes using videos and images, this was coded as “dataset – images and videos” to distinguish them from numerical datasets. The competition level of the athletes was initially coded based on how the authors of the article identified the athletes in the paper (i.e., professional, youth, NCAA, international), which was further categorized into five skill level groups (i.e., Basic, Intermediate, Advanced, Expert, and Mixed) based on the taxonomy proposed by Baker et al. (2015). Athlete age was recorded based on the information provided by the authors (e.g., college aged, high school aged, and mean and standard deviation of the sample). Finally, when coding for sport, most papers focused on a single sport; however, when a paper looked at more than one sport it was coded as “mixed”.

Given that ML applications are a central focus of this review, the research team initially intended to categorize and organize various ML methodologies reported within the included studies. Several approaches were explored, including broad classifications (e.g., supervised vs. unsupervised), specific algorithm types (e.g., decision trees, neural networks, support vector machines), and subject-specific groupings (e.g., injury prediction, image recognition). However, through various attempts and team discussions, it became clear that the diversity and inconsistency in how ML methods were reported, combined with the interdisciplinary nature of this field, made categorization challenging. As a result, any type of classification would not accurately reflect the complexity of the literature. Therefore, methodologies were not grouped into classifications.

### Collating, summarizing, and reporting

Once all articles were finalized and variables extracted, the frequency of each variable was recorded. The findings were then analyzed and discussed within the scope of the review and relative to how they inform future research and practical applications. As is typical for scoping reviews, none of the studies were evaluated for quality and all reporting was based on the direct presentation of the results as reported by the authors of each article.

### Consultation

Consultation with key interest-holders is an optional step in this review process; however, we felt it necessary to ensure we gained as much insight as possible into this ever-growing body of literature. To identify experts, a search was conducted on Web of Science using the key words “machine learning AND sport” to identify individuals who had published five or more articles on the topic. Roughly 100 experts were contacted via email. Of these, 18 responded, the majority turning down our invitation; however, five experts shared articles they believed might meet our inclusion criteria, and one provided a link to their website of relevant publications. In total, 20 articles were submitted, four were already included in our database, and the remaining articles did not meet our inclusion criteria. No experts were available to directly consult during this process.

## Results

**Figure 1:**
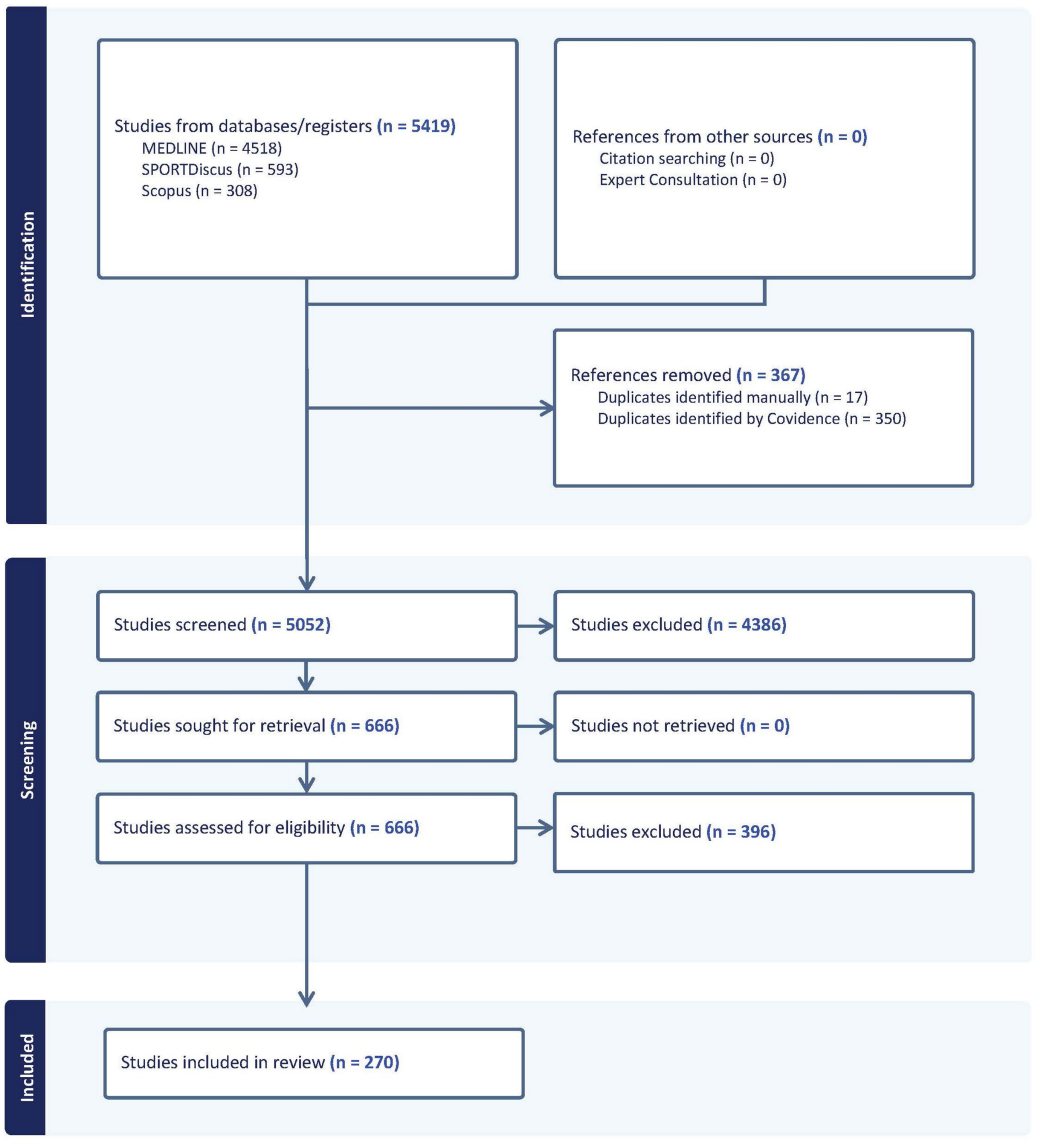
Flow chart of scoping review screening process.

Initially, our search yielded 5,419 articles. After duplicate removal, 5,052 articles remained for title and abstract screening. After the first round of screening 666 articles remained to be assessed for full text eligibility. Once all articles were assessed, conflicts were resolved, and grey areas and tentative inclusions were discussed, 270 articles met our final inclusion criteria. A summary of this process is presented in Figure 1 and our article database can be viewed via this link: http://bit.ly/44Jyk8X.

A total of 44 countries were represented in these articles, with China having the largest number of studies (*n* = 33), representing 12.22%, followed by the United States of America (*n* = 31; 11.48%), Germany (*n* = 27; 10%), Australia (*n* = 26; 9.63%), and Canada (*n* = 15; 5.55%). When looking at the types of samples used across the 270 articles, athlete samples represented 46.26% (*n* = 125) of studies, datasets represented 45.18% (*n* = 122), and dataset images and videos represented 6.66% (*n* = 18).

Forty different sports were represented, including both team (*n* = 176; 65.18%) and individual (*n* = 94; 34.81%) sports. Soccer was the most popular (*n* = 69; 25.55%), followed by samples from mixed sports (*n* = 24; 8.88%), marathon/running (*n* = 22; 8.15%), basketball (*n* = 16; 5.9%), and tennis (*n* = 11; 4.07%). Athlete skill level was classified into five groups, including: (1) Basic (*n* = 19; 7.04%), which accounted for club and regional level sport participation, (2) Intermediate (*n* = 28; 10.37%), which accounted for college/NCAA/Division 1 and state/provincial athletes, (3) Advanced (*n* = 73; 27.04%), which accounted for National level athletes, (4) Expert (*n* = 116; 42.96%), which accounted for International/Olympic and professional athletes, and (5) Mixed (*n* = 23, 8.51%), which contains a combination of two or more levels of athletes. There were also 20 studies (7.40%) that did not fit into this taxonomy, as the athletes’ levels were not specified and were officially coded as “other”. Ages of athletes were not able to be categorized due to inconsistent reporting (e.g., often vague descriptors like ‘college athletes’ were used); however, athletes ranging from youth (aged 12 years) to master’s athletes (aged 88 years) were represented.

A total of 12 broad subject areas were identified. Computer Science made up the largest portion of studies at 41.85% (*n* = 113), followed by Biomechanics (*n* = 46; 17.03%), Sport Psychology (*n* = 42; 15.55%), Physiology (*n* = 24; 8.88%), and Sport Medicine (*n* = 24; 8.88%). When considered relative to the specific subject areas (*n* = 35), Biomechanics (*n* = 46; 17.03%) made up a large portion of the articles, followed by Performance Predictors (*n* = 29; 10.74%), Physiology (*n* = 24; 8.88%), Injury/Concussion (*n* = 24; 8.88%), and Miscellaneous^3^ (*n* = 17; 6.29%).

## Discussion

This scoping review aimed to summarize the evolving application of ML in sport and examine its potential value for athletes, coaches, and sport interest-holders. A secondary objective was to identify key research areas, highlight gaps in the literature, and explore the challenges and opportunities associated with ML technologies. The findings from the 270 included articles reflect a broad and growing interest in ML applications across diverse sporting contexts. The following sections synthesize prominent and emerging research areas, discuss implications for practice and identify priorities for future research.

### General trends

The results of the scoping review demonstrate the diversity of ML applications across sports. Demographically, studies included athletes of all ages, from youth athletes at a club level to elite professional and Olympic level athletes, illustrating the breadth of contexts in which ML is being tested and integrated across various stages of an athlete’s developmental pathway. This demographic diversity was mirrored by the geographic diversity with research conducted across multiple continents, denoting a global interest in these technologies. Among the 40 sports represented, soccer emerged as the most frequently studied (over a quarter of the studies), likely due to its global popularity and the availability of large, publicly accessible datasets from professional leagues and clubs. Within soccer alone, ML has been applied to diverse tasks, such as predicting injury risk using classification-based ML algorithms (Tsilimigkras et al., 2024), talent identification using cross-validated lasso regression (Kelly et al., 2022), and training load monitoring via comparative ML approaches (Vallance et al., 2023). Although team sports seem to be a large focus (nearly two thirds of the studies), potentially due to their easily accessed data, individual sports were also represented in this review (accounting for the remaining third of studies).

### Injury prediction

A large portion of the included studies (17%) focused on injury related applications of ML, including prediction, prevention, diagnosis, and risk assessment, which were investigated through a variety of subject areas (e.g., biomechanics, sport medicine and computer science). This emphasis reflects both the increasing availability of athlete monitoring data and the growing prioritization of athlete health and safety (Bergeron et al., 2015; Carter and Micheli, 2011; Mountjoy et al., 2011; Reardon et al., 2019). Consistent with recent reviews (e.g., Leckey et al., 2025; Van Eetvelde et al., 2021; Wang, 2024), supervised ML models, such as random forest, SVM, and NNs, have been employed to predict injury risk based on variables such as training load, biomechanical data, and injury history. Reviews in this area indicate these models have moderate predictive accuracy (e.g., Area Under the Curve (AUC) values ranged from 0.52 to 0.87 across 11 papers predicting injury risk in Van Eetvelde et al., 2021). To improve model performance, researchers have recommended enhancing data and analysis quality, incorporating more diverse athlete populations, and expanding on variables of investigation. The studies included in our review reinforce these earlier critques (e.g., Hecksteden et al., 2023; Martínez-Gramage et al., 2020; Oliver et al., 2020; Rommers et al., 2020), for example, Hecksteden et al. (2023) analyzed injury data from 88 professional soccer players to evaluate ML’s ability to predict injury occurrence from monitoring and screening data. A gradient boosting model achieved moderate discrimination (AUC = 0.61), and performance remained similar on a holdout test set (i.e., introducing unseen data into the model) (AUC = 0.62), suggesting potential for generalizability beyond the training sample. However, the small number of injuries (*n* = 51) led to variability in predictive accuracy and feature importance across data splits, highlighting the limitations of this smaller data set and underscoring the need for external validation and model refinement.

### Action recognition

ML has also been prominently used in action recognition research, to classify and interpret athletic movements, this is also known as human activity recognition (HAR). Within this subject area, it is worth distinguishing two related, but meaningfully different, areas of inquiry. The first is activity recognition, which classifies discrete movement types, determining, for example whether an athlete is jumping, running, or changing direction (Bulling et al., 2014). The second is form and biomechanical measurement, which, in contrast, focuses on determining subtle differences in how that movement is performed, either within the same individual or between different individuals (Cust et al., 2019). This distinction is important as the first area is concerned with *what* an athlete is doing, while the second is concerned with *how well* or how *consistently* they are doing it. Both approaches are represented in the included literature and share a common methodology, frequently relying on wearable sensors or computer vision to capture movement data.

Perri et al. (2022), for instance, used SVMs and random forests to identify and classify tennis strokes with up to 98% accuracy – this is a clear example of action recognition, where the goal is to correctly label the type of stroke being performed. Similarly, Liu et al. (2024) employed support vector machines (SVM) and decision trees to classify Taekwondo kicks using accelerometer data, demonstrating how wearable technologies can be applied to discrete classification for skill refinement and performance feedback. On the other hand, Ramos Félix et al. (2019) developed SwimBIT, a wearable system using Attitude and Heading Reference System (AHRS) with ML workflows to analyze swim strokes and body dynamics. This study demonstrates a biomechanical measurement approach, where the activity is fixed and the focus shifts to the quality and consistency of movement recognition. While these applications show promise for real-time feedback, skill acquisition, and coaching support, broader validation across diverse sport populations and contexts is necessary.

### Performance prediction and talent identification

ML applications have also been widely used in performance prediction, where models are used to forecast outcomes based on physiological, biomechanical, and contextual data. In swimming, Bayesian networks have been used to optimize relay team composition, revealing that swimmer order (i.e., the position each swimmer will take for the relay) significantly influences performance outcomes (Wu et al., 2021). This information may be helpful to coaches and other support staff in organizing and strategizing team orders. In running, a multitasking deep learning model enabled the classification of performance levels while simultaneously analyzing biomechanical patterns from wearable sensors, enabling real-time performance monitoring (Liu et al., 2020). Gaining this level of precision when measuring performance level may have implications for coaching, injury prevention and determining where an athlete stands in their developmental timeline.

In a similar vein, ML has been used to support talent identification across various sports. Owen et al. (2022) demonstrated that psychosocial traits were as predictive as physical metrics for athlete selection using SVMs and decision trees. In gymnastics, ensemble models and logistic regression were used to identify high potential female athletes early in their development (Pion et al., 2017). Furthermore, Jennings et al. (2024) found that NNs outperformed logistic regression in predicting successful draft outcomes in Australian Rules football. These findings suggest ML can complement traditional scouting methods by integrating diverse data sources and identifying latent predictors of success.

### Outcome prediction

The ability to predict match and game outcomes is a growing area of investigation, offering a theoretical avenue to bring greater certainty to situations that are inherently uncertain. Across sports, researchers have started to adopt more advanced techniques, moving beyond descriptive analyses, to capture the multifaceted nature of sports and performance. For example, a study on elite Australian football utilized ML to analyze a wide range of performance and context indicators, such as player movement, scoring patterns, and environmental conditions to understand their impact and ultimately how these indicators impact the ability to predict match outcomes (Fahey-Gilmour et al., 2019). This approach reflects the growing understanding that match results are based on complex, interacting variables rather than isolated metrics.

Similar trends are evident in other sports, in American football, Beal et al. (2020) conducted a critical comparison of machine learning classifiers to predict NFL game outcomes. Rather than relying on a single model, the authors tested nine algorithms (e.g., decision trees, random forest, and neural network), to determine which most accurately classified wins and losses based on information from 42 features for each team (e.g., points scored, yards gained, offensive plays) and situational factors (e.g., home advantage). In contrast, research on 3 × 3 basketball emphasized contextual variables such as possession structure, defensive rebounds and turnovers and opponent quality (Chen et al., 2024). In this context, machine learning was used to weigh the relative importance of these factors and uncover patterns unique to the fast paced and small sided format of the game. Collectively, these studies demonstrate that although predictive analytics is a shared goal across sports, game and match outcome prediction can no longer be based on a win:loss ratio.

### Emerging and niche applications

Several studies explored novel or less conventional uses of ML, highlighting the versatility of these methods. For instance, Garnica-Caparrós and Memmert (2021) applied explainable ML models to examine gender differences in professional European soccer, identifying distinct patterns in passing intensity, passing behaviour, and spatial occupation between men’s and women’s performances. In endurance sport, Thuany et al. (2023) used XGBoost – a high performance ensemble learning algorithm – to analyze Ironman 70.3 race data, identifying the fastest global racecourse and revealing how course characteristics and environmental factors influenced elite endurance performance. These applications differ meaningfully from areas that currently dominate ML in sport research, such as injury prediction, action recognition, performance and talent identification and predicting match outcomes (Dindorf et al., 2023; Reis et al., 2024). Furthermore, the studies mentioned are consistent with bibliometric analyses of AI and ML research in sport, which has identified the emergence of new research clusters beyond the traditionally studied areas (Dindorf et al., 2023; Reis et al., 2024).

The snapshot of studies included in this review illustrates the breadth of ML applications in sport, highlighting both their promise and limitations. While many models demonstrate promising accuracy and utility, challenges related to data quality, interpretability, and implementation remain. These considerations are particularly important when evaluating how ML technologies influence roles, responsibilities, and experiences of athletes, coaches, and sport interest-holders. Across the 270 studies in this review a consistent gap emerged between technical development and practical utility. Many of the studies reviewed focus on model development and validation, offering limited guidance on real-world implementation. For instance, it remains unclear whether coaches or athletes could meaningfully apply injury prediction or action recognition models without specialist training in data science of biomechanics. Without clear applications for findings or user-friendly interfaces, even the most innovative models risk going unused. This raises issues regarding the purpose and direction of current research, which is often driven more by novelty than practical relevance, underscoring the need for interdisciplinary collaboration to ensure ML tools are designed with end users in mind. Inconsistency in reporting standards also warrants attention. A considerable number of included studies failed to adequately report their populations by age, sex or competitive level, which limits the transferability of findings. For example a model developed and validated on elite adult male athletes may not be applicable in youth, female, or recreational populations. Clearer and more consistent population reporting would substantially improve the field’s ability to identify for whom, and under what conditions, these tools are actually useful.

It is clear that ML technologies are already being implemented in sport, whether or not those affected (e.g., athletes, coaches, and officials) fully understand them. Bridging the gap between technical development and meaningful use will require progress in several areas, such as (a) developing models that are interpretable and explainable by users, (b) building educational resources and training programs to equip coaches, athletes, officials, and administrators to be able to engage and understand these tools, and (c) fostering collaboration between data scientists, sport researchers and practitioners throughout the development process. Ultimately, the goal of this field should not be to model accuracy alone, but whether these tools meaningfully support the people who use them, preserving athlete welfare, enhancing coach decision-making and upholding sport integrity. The question is not only what ML can do, but how, where, and for who it can be meaningfully applied.

### Limitations

Although the research team attempted to make this review as inclusive as possible, there are a few limitations that can be improved upon by future reviews in this area. For example, this review only focused on three databases, and although they were comprehensive in their ability to categorize articles, and focused on sport, it is possible some articles were missed. Furthermore, the present review focused solely on competitive athletes, meaning studies with applications for coaches, referees, judges, and recreational sport were removed. Another limitation was our challenge in categorizing ML applications. It may be helpful for future studies to develop a reporting guideline so that researchers and practitioners, especially those outside of the field, have a better understanding of the methodologies used. Finally, our research team was comprised of only English speakers, and therefore articles not available in English had to be excluded.

## Conclusion

This scoping review revealed the expansive role of ML in sport, with applications spanning athlete monitoring and performance prediction to injury prevention and talent identification. The breadth of research across disciplines and sport types reflects the growing global interest in utilizing ML to enhance sport outcomes. However, despite promising methodological advancements, the practical implementation of ML tools appears limited with more focus on development and validation of these models rather than offering applications for end users, such as coaches, athletes, and sport interest holders. This disconnect highlights the needs for interdisciplinary collaboration and user centered design to ensure ML technologies are both effective and usable in real world sport settings. Importantly, ML should be seen as a tool to support human decision-making (not replace) and should be guided by transparency and a commitment to preserving human elements that define sport performance and experience. Finally, future research should prioritize stakeholder engagement and the development of frameworks to translate these complex models into actionable insights as ML has the power to transform sport.

## Footnotes

1
https://www.covidence.org/

2 Conference proceedings were included in this review as technology sciences and engineering rely heavily on conferences to share their work.

3 Miscellaneous was the subject area given to a number of articles that did not fit into a specific subject area but were still considered important applications of machine learning in the area of sport.
