# Supplementary material for: Machine learning applications in sport: a scoping review
Source: Front Psychol. 2026 May 25;17:1802549. doi: 10.3389/fpsyg.2026.1802549 (PMC13245235; doi:10.3389/fpsyg.2026.1802549)
Supplement: Supplementary file 1 [file Table_1.DOCX]

**MEDLINE**

| **#** | **Searches** | **Results** |
| --- | --- | --- |
| 1 | exp Machine Learning/ | 77451 |
| 2 | exp Deep Learning/ | 23258 |
| 3 | exp Neural Networks, Computer/ | 72821 |
| 4 | Athletes/ | 22775 |
| 5 | (sport or sports or sporting or archery or athletics or badminton or baseball or basketball or biathlon or bobsleigh or canoe or rowing or softball or handball or volleyball or gymnastics or skiing or curing or cycling or diving or equestrian or fencing or figure skating or football or futsal or golf or hockey or ice hockey or judo or karate or lacrosse or luge or swimming or pentathlon or Nordic combined or speed skating or rugby or sailing or shooting or skateboarding or skeleton or mountaineering or snowboard or squash or surfing or table tennis or taekwondo or tennis or trampoline or triathlon or polo or weightlifting or wrestling or wushu).tw,kf. | 361075 |
| 6 | (machine learning or deep learning or neural networks).tw,kf. | 220480 |
| 7 | (athlete or athletes or athletics).tw,kf. | 76713 |
| 8 | (Machine Learning or Deep Learning or Neural Networks, Computer or (machine learning or deep learning or neural networks)).tw,kf. | 220480 |
| 9 | sports/ or baseball/ or basketball/ or bicycling/ or boxing/ or cricket sport/ or football/ or golf/ or gymnastics/ or hockey/ or martial arts/ or mountaineering/ or racquet sports/ or rugby/ or running/ or skating/ or snow sports/ or soccer/ or team sports/ or volleyball/ or walking/ or water sports/ or weight lifting/ or wrestling/ | 151288 |
| 10 | (Athletes or (sport or sports or sporting or archery or athletics or badminton or baseball or basketball or biathlon or bobsleigh or canoe or rowing or softball or handball or volleyball or gymnastics or skiing or curing or cycling or diving or equestrian or fencing or figure skating or football or futsal or golf or hockey or ice hockey or judo or karate or lacrosse or luge or swimming or pentathlon or Nordic combined or speed skating or rugby or sailing or shooting or skateboarding or skeleton or mountaineering or snowboard or squash or surfing or table tennis or taekwondo or tennis or trampoline or triathlon or polo or weightlifting or wrestling or wushu) or (athlete or athletes or athletics) or (sports or baseball or basketball or bicycling or boxing or cricket sport or football or golf or gymnastics or hockey or martial arts or mountaineering or racquet sports or rugby or running or skating or snow sports or soccer or team sports or volleyball or walking or water sports or weight lifting or wrestling)).tw,kf. | 551281 |
| 11 | ((Machine Learning or Deep Learning or Neural Networks, Computer or (machine learning or deep learning or neural networks)) and (Athletes or (sport or sports or sporting or archery or athletics or badminton or baseball or basketball or biathlon or bobsleigh or canoe or rowing or softball or handball or volleyball or gymnastics or skiing or curing or cycling or diving or equestrian or fencing or figure skating or football or futsal or golf or hockey or ice hockey or judo or karate or lacrosse or luge or swimming or pentathlon or Nordic combined or speed skating or rugby or sailing or shooting or skateboarding or skeleton or mountaineering or snowboard or squash or surfing or table tennis or taekwondo or tennis or trampoline or triathlon or polo or weightlifting or wrestling or wushu) or (athlete or athletes or athletics) or (sports or baseball or basketball or bicycling or boxing or cricket sport or football or golf or gymnastics or hockey or martial arts or mountaineering or racquet sports or rugby or running or skating or snow sports or soccer or team sports or volleyball or walking or water sports or weight lifting or wrestling))).tw,kf. | 4518 |

**SCOPUS**

( TITLE-ABS-KEY ( ( machine AND learning OR deep AND learning OR neural AND networks ) ) ) AND ( ( TITLE-ABS-KEY ( ( sport OR sports OR sporting OR archery OR athletics OR badminton OR baseball OR basketball OR biathlon OR bobsleigh OR canoe OR rowing OR softball OR handball OR volleyball OR gymnastics OR skiing OR curing OR cycling OR diving OR equestrian OR fencing OR figure AND skating OR football OR futsal OR golf OR hockey OR ice AND hockey OR judo OR karate OR lacrosse OR luge OR swimming OR pentathlon OR nordic AND combined OR speed AND skating OR rugby OR sailing OR shooting OR skateboarding OR skeleton OR mountaineering OR snowboard OR squash OR surfing OR table AND tennis OR taekwondo OR tennis OR trampoline OR triathlon OR polo OR weightlifting OR wrestling OR wushu ) ) ) OR ( TITLE-ABS-KEY ( ( athlete OR athletes OR athletics ) ) ) )

308 articles

**SportDicsus**

| Thu, October 17, 2024 1:14:47 PM |
| --- |

| **#** | **Query** | **Limiters/Expanders** | **Last Run Via** | **Results** |
| --- | --- | --- | --- | --- |
| S6 | TI ( (S4 AND S5) ) OR AB ( (S4 AND S5) ) OR KW ( (S4 AND S5) ) | Expanders - Apply equivalent subjects  Search modes - Proximity | Interface - EBSCOhost Research Databases  Search Screen- Advanced Search  Database - SPORTDiscus | 593 |
| S5 | TI ( (S1 OR S2 OR S3) ) OR AB ( (S1 OR S2 OR S3) ) OR KW ( (S1 OR S2 OR S3) ) | Expanders - Apply equivalent subjects  Search modes - Proximity | Interface - EBSCOhost Research Databases  Search Screen- Advanced Search  Database - SPORTDiscus | 1,236,102 |
| S4 | TI ( (machine learning OR deep learning OR neural networks) ) OR AB ( (machine learning OR deep learning OR neural networks) ) OR KW ( (machine learning OR deep learning OR neural networks) ) | Expanders - Apply equivalent subjects  Search modes - Proximity | Interface - EBSCOhost Research Databases  Search Screen- Advanced Search  Database - SPORTDiscus | 2,271 |
| S3 | TI ( (athlete OR athletes OR athletics) ) OR AB ( (athlete OR athletes OR athletics) ) OR KW ( (athlete OR athletes OR athletics) ) | Expanders - Apply equivalent subjects  Search modes - Proximity | Interface - EBSCOhost Research Databases  Search Screen- Advanced Search  Database - SPORTDiscus | 290,641 |
| S2 | TI ( (sport OR sports OR sporting OR archery OR athletics OR badminton OR baseball OR basketball OR biathlon OR bobsleigh OR canoe OR rowing OR softball OR handball OR volleyball OR gymnastics OR skiing OR curing OR cycling OR diving OR equestrian OR fencing OR figure skating OR football OR futsal OR golf OR hockey OR ice hockey OR judo OR karate OR lacrosse OR luge OR swimming OR pentathlon OR Nordic combined OR speed skating OR rugby OR sailing OR shooting OR skateboarding OR skeleton OR mountaineering OR snowboard OR squash OR surfing OR table tennis OR taekwondo OR tennis OR trampoline OR triathlon OR polo OR weightlifting OR wrestling OR wushu) ) OR AB ( (sport OR sports OR sporting OR archery OR athletics OR badminton OR baseball OR basketball OR biathlon OR bobsleigh OR canoe OR rowing OR softball OR handball OR volleyball OR gymnastics OR skiing OR curing OR cycling OR diving OR equestrian OR fencing OR figure skating OR football OR futsal OR golf OR hockey OR ice hockey OR judo OR karate OR lacrosse OR luge OR swimming OR pentathlon OR Nordic combined OR speed skating OR rugby OR sailing OR shooting OR skateboarding OR skeleton OR mountaineering OR snowboard OR squash OR surfing OR table tennis OR taekwondo OR tennis OR trampoline OR triathlon OR polo OR weightlifting OR wrestling OR wushu) ) OR KW ( (sport OR sports OR sporting OR archery OR athletics OR badminton OR baseball OR basketball OR biathlon OR bobsleigh OR canoe OR rowing OR softball OR handball OR volleyball OR gymnastics OR skiing OR curing OR cycling OR diving OR equestrian OR fencing OR figure skating OR football OR futsal OR golf OR hockey OR ice hockey OR judo OR karate OR lacrosse OR luge OR swimming OR pentathlon OR Nordic combined OR speed skating OR rugby OR sailing OR shooting OR skateboarding OR skeleton OR mountaineering OR snowboard OR squash OR surfing OR table tennis OR taekwondo OR tennis OR trampoline OR triathlon OR polo OR weightlifting OR wrestling OR wushu) ) | Expanders - Apply equivalent subjects  Search modes - Proximity | Interface - EBSCOhost Research Databases  Search Screen- Advanced Search  Database - SPORTDiscus | 1,099,223 |
| S1 | DE "AERONAUTICAL sports" OR DE "AQUATIC sports" OR DE "BASEBALL" OR DE "COLLEGE sports" OR DE "COMBAT sports" OR DE "CONTACT sports" OR DE "CROSS-training (Sports)" OR DE "ENDURANCE sports" OR DE "GYMNASTICS" OR DE "HOCKEY" OR DE "INDIVIDUAL sports" OR DE "ROLLER skating" OR DE "SHOOTING (Sports)" OR DE "SKATEBOARDING" OR DE "SOFTBALL" OR DE "TEAM sports" OR DE "ATHLETES" OR DE "BADMINTON players" OR DE "BASEBALL players" OR DE "BASKETBALL players" OR DE "BOBSLEDDERS" OR DE "BODYBUILDERS" OR DE "BOWLERS" OR DE "BOXERS (Sports)" OR DE "CANOEISTS" OR DE "CRICKET players" OR DE "CURLERS (Athletes)" OR DE "CYCLISTS" OR DE "DEFENSIVE players" OR DE "ELITE athletes" OR DE "FENCERS" OR DE "FOOTBALL players" OR DE "GLADIATORS" OR DE "GOLFERS" OR DE "GYMNASTS" OR DE "HANDBALL players" OR DE "HOCKEY players" OR DE "JAI alai players" OR DE "KABADDI players" OR DE "LACROSSE players" OR DE "MARTIAL artists" OR DE "MOUNTAINEERS" OR DE "NETBALL players" OR DE "OFFENSIVE players" OR DE "ORIENTEERS" OR DE "ROWERS" OR DE "RUGBY football players" OR DE "RUNNERS (Sports)" OR DE "SKATERS" OR DE "SKIERS" OR DE "SNOWBOARDERS" OR DE "SOCCER players" OR DE "SOFTBALL players" OR DE "SQUASH players" OR DE "SURFERS" OR DE "SWIMMERS" OR DE "TABLE tennis players" OR DE "TEAM handball players" OR DE "TENNIS players" OR DE "TRACK & field athletes" OR DE "TRIATHLETES" OR DE "VOLLEYBALL players" OR DE "WATER polo players" OR DE "WEIGHT lifters" OR DE "WRESTLERS" OR DE "SPORTS" | Expanders - Apply equivalent subjects  Search modes - Proximity | Interface - EBSCOhost Research Databases  Search Screen- Advanced Search  Database - SPORTDiscus | 463,490 |
